# Alpha-band power increases in posterior brain regions in attention deficit hyperactivity disorder after digital cognitive stimulation treatment: randomized controlled study

**DOI:** 10.1093/braincomms/fcac038

**Published:** 2022-02-17

**Authors:** Ignacio de Ramón, Javier Pacios, Rafael Medina, Jaime Bouhaben, Pablo Cuesta, Luis Antón-Toro, Javier Quintero, Antoni Ramos Quiroga, Fernando Maestú

**Affiliations:** 1 Sincrolab, Ltd, Madrid 28033, Spain; 2 Department of Experimental Psychology, Complutense University of Madrid, Madrid 28040, Spain; 3 Department of Radiology Rehabilitation and Physiotherapy, Complutense University of Madrid, Madrid 28040, Spain; 4 Department of Psychiatry, University Hospital Infanta Leonor, Madrid 28031, Spain; 5 Department of Psychiatry, Hospital Vall d´Hebron, Barcelona 08035, Spain

**Keywords:** magnetoencephalography, power spectrum, alpha-band power, attention deficit and hyperactivity disorder, cognitive stimulation treatment

## Abstract

The changes triggered by pharmacological treatments in resting-state alpha-band (8–14 Hz) oscillations have been widely studied in attention deficit hyperactivity disorder. However, to date, there has been no evidence regarding the possible changes in cognitive stimulation treatments on these oscillations. This paper sets out to verify whether cognitive stimulation treatments based on progressive increases in cognitive load can be effective in triggering changes in alpha-band power in attention deficit hyperactivity disorder. With this objective, we compared a cognitive stimulation treatment (*n* = 12) to a placebo treatment (*n* = 14) for 12 weeks (36 sessions of 15 min) in child patients (8–11 years old) with attention deficit hyperactivity disorder. Two magnetoencephalographic recordings were acquired for all participants. In order to extract the areas with changes in alpha power between both magnetoencephalographic recordings, the differences in the power ratio (pre/post-condition) were calculated using an analysis of covariance test adjusted for the age variable. The results show an increase in the post-treatment alpha power in the experimental group versus the placebo group (*P* < 0.01) in posterior regions. In addition, these changes were related to measures of attention, working memory and flexibility. The results seem to indicate that cognitive stimulation treatment based on progressive increases in cognitive load triggers alpha-band power changes in children diagnosed with attention deficit hyperactivity disorder in the direction of their peers without this disorder.

## Teaser

The magnetoencephalography study by Medina *et al.* reports a game-like cognitive intervention for children with attention deficit hyperactivity disorder that can induce an enhancement in alpha power over the posterior regions of the brain. Moreover, this alpha power enhancement negatively correlates with attention deficit symptoms.

## Graphical Abstract

**Graphical Abstract:**
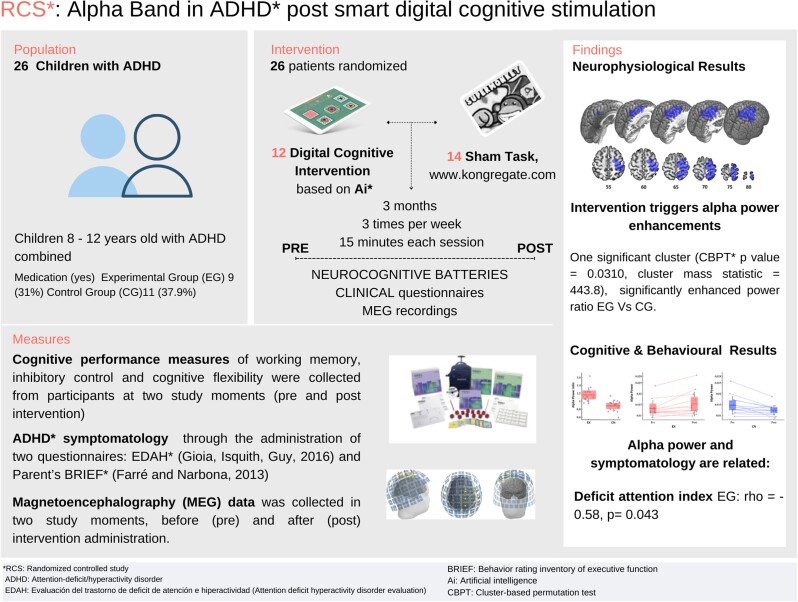

## Introduction

Oscillatory power in the range of the alpha band (8–14 Hz) is one of the most stable brain function indices^1,2^ and with greater test–retest reliability.^3^ Resting-state alpha-band oscillations have been widely studied in various psychiatric disorders. These studies indicate that patients diagnosed with schizophrenia, obsessive-compulsive disorder, autism, depression or attention deficit hyperactivity disorder (ADHD) present changes in the different dynamics of this frequency band (for an in-depth review, see Newson and Thiagarajan^4^). Specifically, oscillatory activity in the alpha band seems to have an intimate relationship with cognitive processes such as working memory maintenance^5^ or inhibitory control,^6^ both of which are known to be affected in ADHD.^7^

Previous studies have reported decreases in the relative alpha-band power in children diagnosed with ADHD. This effect has been located mainly in central and posterior brain regions.^4,8–15^ Moreover, this effect has been shown to be modulated by individual performance in attention and inhibitory control tasks in children diagnosed with ADHD.^16,17^ Interestingly, this reduction seems to be highly heritable between individuals of the same family,^18^ and especially from parents to their children.^19^

Both pharmacological studies using stimulant drugs and non-pharmacological interventions (i.e. physical exercise, Frutos-Lucas *et al*.^20^) have been developed to test their potentiality to reverse the changes in alpha power in this disorder (for an in-depth review, see Kirkland and Holton^21^). However, the effect of cognitive stimulation on abnormal alpha oscillatory dynamics has received scarce attention in the field of ADHD, even though it is among the most important therapeutic options, accumulating evidence on its efficacy to reduce the symptomatology and the associated cognitive disturbances.^22–27^ To date and as far as our literature review shows, only two studies have addressed this important issue. Johnstone *et al*.^28^ found an increase in beta-band power (12–30 Hz) after a computer-based cognitive training and Deiber *et al*.^29^ showed an enhancement of alpha-band power after neurofeedback treatment. Notwithstanding, evidence drawn from studies looking at the effect of different interventions in other neuropsychiatric conditions has shown that therapeutic changes are also accompanied by increases in oscillatory power within the alpha band. For instance, patients with alcoholism seem to experience an enhancement of alpha power after completing a cognitive-behavioural therapy.^30^

Thus, the motivation of the present study was to evaluate the therapeutic effect of a cognitive intervention in children diagnosed with ADHD, both at the clinical-cognitive and the neurophysiological level. To achieve our objective, we implemented a digital cognitive stimulation treatment in a group of children diagnosed with ADHD. The specific intervention was based on the progressive increase in cognitive load, since this approach is thought to be the most effective one for ameliorating clinical and neuropsychological symptoms.^24^ Indeed, increases in cognitive load have been shown to induce a transition from ‘highly modular architecture to promote more integrated information processing’, enhancing long-distance connections.^31–33^ Critically, in the present study increases in cognitive load were supervised by machine learning algorithms. This new way of modulating the increase in cognitive load makes it possible to adapt, in real time, the tasks within the training to the most appropriate challenging level for each patient. Indeed, this type of algorithm has already been shown to be effective in improving cognitive processes affected in ADHD patients.^34^

Based on the studies reviewed we hypothesized that ADHD children in the experimental group should exhibit a significant increase in alpha power (8–14 Hz) after completing a 12-week intervention, in comparison with those in the sham group. Although previous studies suggest that alpha power effects are primarily located over parietal regions, the spatial precision of magnetoencephalography (MEG) is lower than that provided by functional magnetic resonance, particularly in studies in which individual anatomical images are not available for forward modelling (see the ‘Source Reconstruction’ section). Thus, we chose not to define an *a priori* hypothesis regarding the topography of the effect, and maintain our analysis within a whole-brain perspective.

## Materials and methods

### Participants

*A priori* sample size was estimated to detect a standardized mean difference of 0.64 SD in Commission score from Conners' Continuous Performance Test (CPT-III),^35^ with significance level of *α* = 0.05 and power of 0.8 (1 − *β* = 0.8). The calculation procedure follows sample size estimation for a two-tailed, two-sample mean difference with a correction factor for repeated measures.^36^ This analysis suggested a required sample size of *N* = 56 (*n* = 28). Unfortunately, COVID-19 crisis and its consequences in Spain (since March 2020), forced the Sponsor and the PI to stop the recruitment procedures due to the difficulties in order to assure protocol compliance in 2020. Therefore, the last enrolled participant ended study procedures in February 2020. Finally, a sample of 41 participants (8–11 years) diagnosed with ADHD of the combined presentation was recruited. Participants were enrolled from schools, hospitals and associations from the Community of Madrid. All of them are Spanish native speakers. ADHD diagnosis was performed by a collegiate health professional according to DSM-IV or V criteria. A team of expert neuropsychologists ensured that individuals willing to participate met inclusion criteria.^37^

Inclusion criteria were: (i) aged 8–11; (ii) diagnosis of ADHD-combined by an authorized professional (Chartered psychiatrists at the Medical College); (iii) have stopped taking ADHD medication 3 days before each visit day; as, according to the technical sheet of the drug *methylphenidate* (CONCERTA®), it has a half-life of 3.5 h (90% is excreted in urine and 1–35 in faeces as a metabolite at 48–96 h); (iv) maintain the same level of medication during the at-home intervention period and (v) compliance with intervention protocol.

Exclusion criteria were: (i) begin or abandon behavioural therapies or psychoactive drugs during the at-home intervention period; (ii) motor difficulties which make the use of the device impossible; (iii) use of psychoactive drugs which may be a confounding factor (such as benzodiazepines); (iv) presence or suspicion of substance abuse for the last 6 months; (v) presence of blindness or uncorrected visual acuity difficulties and (vi) have any additional psychological diagnosis.

From this initial pool, 40 of them received, exclusively, either an experimental neuropsychological digital treatment, *KAD_SCL_01®* (Experimental group) or a sham control intervention (Control group). MEG analyses, as described in the proper sections, were applied to a final sample of 26 participants (Experimental, *n* = 12; Control, *n* = 14). Eleven participants were considered as analyses' dropouts due to meet exclusion criteria (incompliance or abandonment of the intervention protocol). Three participants were excluded from the analysis plan due to invalid MEG data (Table 1).

**Table 1:** Demographic and baseline cognitive characteristics in experimental and control groups

Characteristic	Experimental mean (SD) or proportion	Control mean (SD) or proportion	*t*/*χ*	*P*-value
Age	9.2 (1.21)	9.71 (1.33)	1.09	0.286^a^
Sex (males)	12 (46.15%)	13 (50%)	0.005	0.941^b^
Medication (yes)	9 (31%)	11 (37.9%)	1.17	0.28^b^
Other psychological treatment (yes)	4 (13.8%)	3 (10.3%)	0.11	0.742^b^
Visuospatial working memory	5.8 (2.14)	5.64 (1.94)	−0.65	0.52^a^
Flexibility	3.2 (2.30)	4.64 (2.61)	72.5	0.16^a^
Inhibitory control	53.87 (8.37)	49.79 (7.53)	1.72	0.09^a^
Attention	57.46 (7.69)	52.85 (8.06)	1.5	0.12^a^
Attention deficit symptoms	97.82 (2.16)	92.57 (13.35)	1.08	0.29^a^
Hyperactivity deficit symptoms	93.6 (9.71)	91.28 (11.33)	0.61	0.54^a^
Global symptoms composite score	97.86 (3.60)	95.71 (6.85)	−1.04	0.30^a^
Behaviour regulation composite score	89.86 (8.95)	88.57 (16.91)	−0.25	0.80^a^
Executive function composite score	94.4 (3.24)	92.14 (7.72)	−1.01	0.32^a^

^a^ *p*-values are from a *t*-test (between-subject, two-tailed).

^b^ *p*-values are from a *χ*^2^ test (two-tailed).

### Experimental design and procedure

The present research is a randomized controlled study that follows a 2 × 2 mixed factorial design with two independent arms and two repeated measures. The study was approved by the Ethics Committee from San Carlos University Hospital (Madrid, Community of Madrid) and the procedure was performed following the Helsinki Declaration^38^ and national and European Union regulations. The study was pre-registered in the ISRCTN database with the following number: *ISRCTN71041318.* All participant's legal guardians signed an informed consent prior to any activity within the study. Informed consent model was approved by the Ethics Committee from San Carlos University Hospital (Madrid, Community of Madrid).

Participants were allocated in one of the two independent arms (Experimental or Control) according to a randomization schema with 1:1 ratio. Both groups carried out an at-home digital intervention for 12 weeks. The complete intervention was divided into training sessions. Intervention protocol established three 15-min training sessions per week for 12 weeks, giving an amount of 36 training sessions. Compliance with intervention protocol meant at least the completion of at least an 80% of the scheduled training sessions (see Supplementary material 2 for a complete descrition of subjects dropouts). Participants did not receive specific information about the group they were assigned to. Moreover, data analysis was completed by scientists who were blinded to the group label of the subject.

The experimental arm performed a neuropsychological digital treatment, *KAD_SCL_01®*. This is a digital software with an integrated artificial intelligence (AI) engine which generates a complete cognitive stimulation intervention based on 14 therapeutic videogames (see Supplementary material 3 for a complete description). The AI engine, based on case-based reasoning (CBR) algorithms, supervises the difficulty parameters of therapeutic games, increasing or decreasing them at each training session according to the individual's performance on previous sessions (see Supplementary material 4 for a complete description).

The Control arm was attached to a sham control intervention composed of three commercial videogames, whose design was not aimed to cognitive rehabilitation or stimulation, according to Mishra *et al*.^39^ criteria. Sham control videogames are available at the open-access platform *Kongregate* (www.kongregate.com).

Cognitive performance measures of working memory, inhibitory control and cognitive flexibility were collected from participants at two study moments (pre- and post-intervention). These variables were taken from standardized neuropsychological tests: Conners' CPT-III,^40^ Corsi Block Tapping Test^41^ and Card Classification test.^42^ In addition, clinical data about ADHD symptomatology were collected from participants' legal guardians through the administration of two questionnaires: EDAH^43^ and Parent's Behavior rating inventory of executive function.^44^

An extended description of the study methodology and procedure is extensively reported in Supplementary material 1.

### MEG recordings

MEG data were collected in two study moments, before (pre) and after (post) intervention administration. Data were acquired using a whole-head Elekta-Neuromag MEG system with 306 channels (Elekta AB, Stockholm, Sweden) at the Center for Biomedical Technology (Madrid, Spain). MEG data were collected at a sampling frequency of 1000 Hz and online band-pass filtered between 0.1 and 330 Hz.

Each data collection (pre and post) consisted of two 5-min resting-state conditions: first with eyes open and next with eyes closed. Before or after MEG data collection, participants underwent a neuropsychological assessment aimed to measure cognitive performance (see Supplementary material 1 for a complete description). The order in which neuropsychological assessment and MEG records were administered was at random. The two resting-state conditions were collected consecutively.

Participants were accommodated inside the magnetically shielded room where MEG engine is placed. Each subject's head shape was defined relative to three anatomical locations (nasion and bilateral preauricular points) using a 3D digitizer (Fastrak, Polhemus, VT, USA) and head motion was tracked through four head position indicator (HPI) coils attached to the scalp. These HPI coils continuously monitored the subjects' head movements, while eye movements were monitored by a vertical electrooculogram (EOG) assembly composed of a pair of bipolar electrodes.

General instructions for participants during MEG recording were to be as quiet, still and relaxed as they were able to and to not move the head outside MEG's helmet. For eyes closed resting-state conditions, participants were also asked for staying 5 min with their eyes closed. Ambient lighting was lowered inside the room in order to favour eyes closed keeping. For eyes open resting state, a screen was placed opposite to the participant, projecting a 3 cm black *plus* sign in a white bottom. Participants were asked for looking at the black *plus sign* for 5 min. Although these two conditions were recorded, MEG analyses were only performed over eyes closed resting-state records.

### Pre-processing and power calculations

Raw recording data were first introduced to Maxfilter software (v 2.2, correlation threshold = 0.9, time window = 10 s) to remove external noise using the temporal extension of the signal-space separation method with movement compensation.^45^ Then, magnetometers data^46^ were automatically examined to detect ocular, muscle and jump artefacts using Brainstorm software,^47^ which were visually confirmed by an MEG expert. The remaining artefact-free data were sectioned into 4 s segments. Afterwards, signal-space projection analysis-based procedure was applied to remove heart magnetic field artefacts and EOG components. Only those recordings with at least 20 clean segments (80 s of brain activity) were utilized in subsequent analyses. MEG clean time series were band-pass filtered (0.5 s padding) between 2 and 45 Hz.

The power spectrum of each grid's node was computed by means of Fast Fourier Transform using Hanning tapers with 0.25 Hz smoothing. For each node, relative power was calculated by normalizing by total power over the 1.5–45 Hz range. The alpha-band average power was computed by averaging across all frequency steps (25 in total) within the alpha-band interval (8–14 Hz). The source template with 2459 nodes in a 10 mm spacing grid was segmented into 78 regions of the Automated Anatomical Labeling (AAL^48^) atlas, excluding the cerebellum, basal ganglia, thalamus and olfactory cortices. Those 78 regions of interest included 1202 of the original 2459 nodes. Trials were averaged across subjects ending up with a source-reconstructed power matrix of 1202 nodes × 26 participants. Finally, the power ratio (post-condition/pre-condition) was calculated to assess the change between the two conditions of the follow-up.

### Source reconstruction

As no individual anatomy was available, source reconstruction was carried out using a template head model. The head model consisted of a single layer representing the inner skull interface, generated from the union of tissues grey matter, white matter and cerebrospinal fluid in the Montreal Neurological Institute (MNI) brain. The source model was defined as a regular grid of 10 mm spacing defined in MNI space, and only those sources falling in an area defined as cortical in the AAL atlas were considered, resulting in 1202 source positions. The scalp of the MNI template was linearly transformed to the individual head shape using an affine transformation generated with an iterative algorithm, and the same transformation was applied to the head and source models. The lead field was calculated using a single shell model.^49^ Sources time series were reconstructed using a Linearly Constrained Minimum Variance beamformer,^50^ using the trial-average covariance matrix and a regularization factor of 5% of the average sensor power.

### Statistical analyses

The power (post-condition/pre-condition) ratio was calculated to assess the change between the two conditions of the follow-up. These values were used to extract the brain region that better differentiate both groups over time. The assessment of significant group power differences was addressed relying on the cluster-based permutation test (CBPT).^51,52^ The methodology started by testing power ratio differences between groups per each pair of nodes using an analysis of covariance (ANCOVA) test while adjusting for the effects of age. The resulting matrix of *F*-statistics (with the same dimension than the original power matrix), was binarized by thresholding the matrix using a critical value computed with a *P*-value = 0.01. This binary matrix was split into two matrices attending to the sign of the differences between groups. Clusters were built by grouping those spatially adjacent nodes that systematically showed significant between-groups differences. Importantly, all nodes within a cluster must have shown the same sign of the between-groups differences, thus indicating that the cluster might be deemed as a functional unit. Only clusters involving at least 1% of the nodes (i.e. a minimum of 12 nodes) were considered. Cluster-mass statistics were assessed through the sum of all *F*-values across all nodes. Then, to control for multiple comparisons, the entire analysis pipeline was repeated 5000 times after shuffling the original group's labels. At each repetition, the maximum statistic of the surrogate clusters was kept, creating a maximal null distribution that would ensure control of the family-wise error rate at the cluster level. Cluster-mass statistics on each cluster in the original data set was compared with the same measure in the randomized data. The CBPT *P*-value represented the proportion of the permutation distribution with cluster-mass statistic values greater or equal than the cluster-mass statistic value of the original data.

Only those clusters that survived the CBPT at *P* < 0.05 or below were considered for the subsequent analyses as potential ‘MEG markers’. As descriptive values, and surrogate effect size, for each significant cluster, we computed the average power (across all nodes that belong to the cluster) for the power ratio, and the power of both, pre and post, conditions. These values were used as MEG marker values for the subsequent Spearman correlation analysis with measures of cognitive performance traits (post-condition/pre-condition) ratio that are known to be affected in ADHD. In addition, we computed pairwise statistics, between and within-groups, for these averaged power values using ANCOVA with age as covariate. Statistical analyses were carried out using MATLAB R2020b (Mathworks Inc.).

### Ethics and trial registration

The study was approved by the Ethics Committee from San Carlos University Hospital (Madrid, Community of Madrid) and the procedure was performed following the Helsinki Declaration^38^ and national and European Union regulations.

The study was pre-registered in the ISRCTN database with the following number: *ISRCTN71041318*.

### Data availability

The data that support the findings of this study are available from the corresponding author, upon request. All the algorithms used in the present paper are reported in the ‘Materials and methods’ section.

## Results

**Figure 1:**
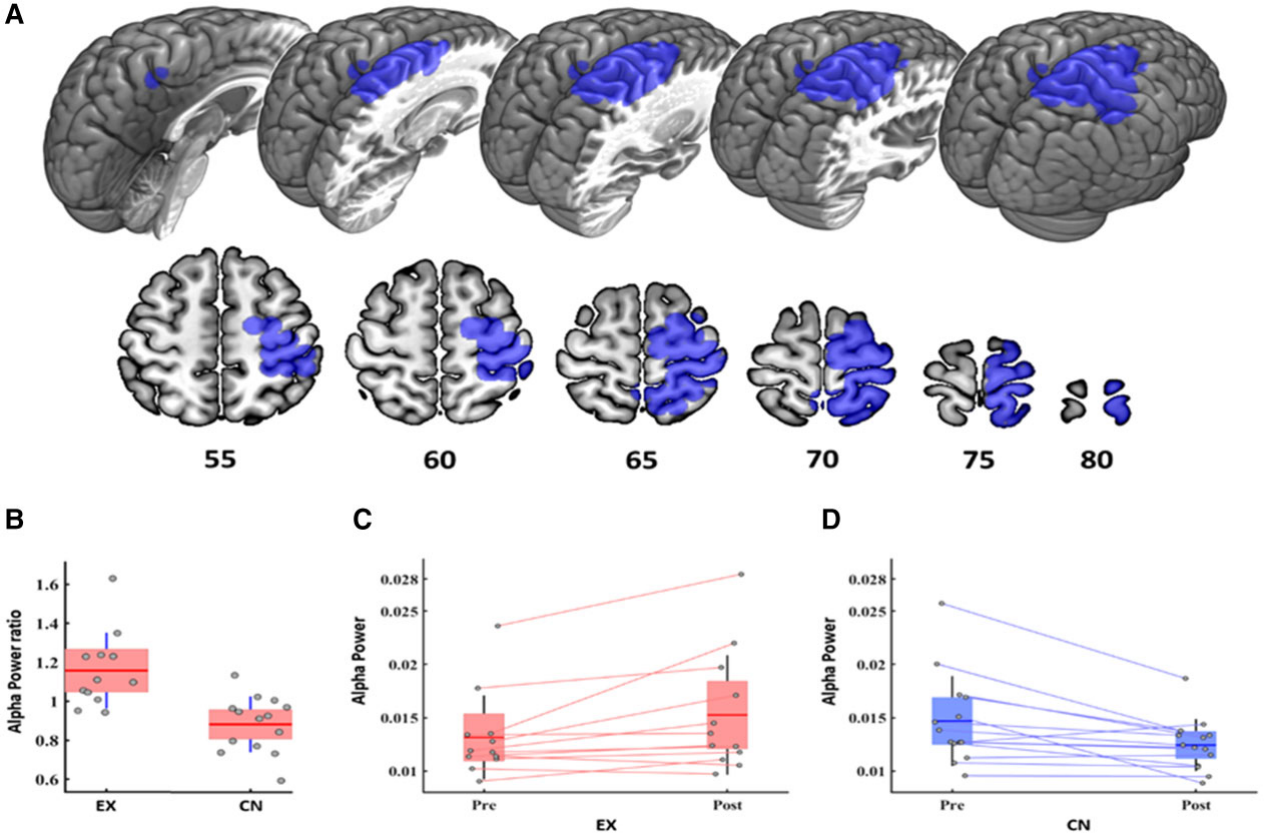
**Between-group changes in power ratio (post/pre) in alpha band**. (**A**) Regions which compose the significant cluster (CBPT statistics; cluster-mass statistic = 443.8; *P-*value = 0.0310) at alpha power band (8–12 Hz): right post-central gyrus, right pre-central gyrus, right superior frontal gyrus, right superior parietal gyrus, right precuneus, right supplementary motor area and right paracentral lobule. (**B**) Descriptive boxplot for the cluster average power ratio (post/pre) in the Experimental and Control group [ANCOVA test between groups with age (*P-*value = 0.0004, *F*-statistic = 17.5)]. (**C** and **D**) Pre- and post-alpha power values per intervention group: Experimental (**C**) and Control (**D**) Wilcoxon between-conditions test for the EX group (*P-*value = 0.0093, signed rank-statistic = 7) and for the CN group (*P-*value = 0.0052, signed rank-statistic = 95).

The power ratio was used to determine the brain region with a significant difference between both groups considering the information of both MEG recordings. The result showed one significant cluster (CBPT *P-*value = 0.0310, cluster-mass statistic = 443.8), mainly comprising posterior regions of the brain (see Fig. 1A and Table 2), with a significantly enhanced power ratio in the Experimental participants when compared with the Control group. We computed the average strength of the nodes contained in the cluster as a surrogate effect size and carried out a new ANCOVA Test with age to quantify it. The values obtained of the differences at the cluster level were *P-*value < 0.0004 and *F*-statistic 17.5. Since the average power ratio of the Experimental group was found to be higher than 1, this result implied that the occipital alpha power was increased along time in these patients (see Fig. 1B and C). On the contrary, the Control patients showed a power ratio below 1, indicating that for those patients the occipital alpha power decreased over time (see Fig. 1B and D). As it can be seen in Fig. 1C and D, the individual trajectories between both MEG recordings showed that most participants behaved in a similar group manner. When addressing these evolution pathways with an additional Wilcoxon between-conditions test, we found significant differences between sessions for both groups: pre versus post within EX group *P*-value = 0.0093, signed rank-statistic = 7; and pre versus post within CN group *P-*value = 0.0052, signed rank-statistic = 95. In addition, we explored the existence of significant differences between-groups at each condition using ANCOVA between-groups test with age as covariate. We did not find significant differences between both groups (ANCOVA test with age as covariate, *P* > 0.1) neither within the pre- nor within the post-conditions.

**Table 2:** AAL ROIs^a^ that were partially captured by the significant cluster

ROI	% of ROI occupied	% of cluster within the ROI
Right postcentral gyrus	39.39	34.21
Right precentral gyrus	29.63	21.05
Right superior frontal gyrus	19.35	15.79
Right superior parietal gyrus	22.22	10.53
Right precuneus	14.29	7.89
Right supplementary motor area	14.29	5.26
Right paracentral lobule	25	5.26

^a^ Regions of interest (ROIs) from the Anatomical Labeling atlas (AAL) that are part of the significant cluster where physical activity correlates with power in the alpha band. It shows, as well, the percentage of each ROI that is captured by that cluster.

Finally, the average power ratio value of the significant cluster was used for new correlation analyses in both subgroups. Alpha power ratio in posterior brain regions correlated with the cognitive performance ratio measure of visuospatial working memory in the overall sample (*ρ* = 0.63, *P* = 0.001) and, in the Sham control (*ρ* = 0.69, *P* = 0.006). The deficit attention symptoms index of the Evaluación del Trastorno por Déficit de Atención con Hiperactividad (Attention deficit hyperactivity disorder evaluation) also correlated in the experimental group (*ρ* = −0.58, *P* = 0.043). Furthermore, flexibility correlated in the overall sample (*ρ* = 0.45, *P* = 0.025). No other correlations were significant. The complete set of correlations is displayed in Table 3.

**Table 3:** Spearman correlations between average power ratio (post/pre) and cognitive and clinical outcomes

Outcome	Overall sample		Experimental		Control	
	*ρ*	*P*-value	*ρ*	*P*-value	*ρ*	*P*-value
Visuospatial working memory	0.63	0.001**	0.29	0.36	0.69	0.006**
Inhibitory control	−0.19	0.34	0.25	0.43	0.13	0.64
Attention	−0.08	0.69	0.43	0.16	0.05	0.87
Flexibility	0.45	0.025*	0.35	0.29	0.38	0.18
Attention deficit symptoms	−0.16	0.43	−0.58	0.046*	−0.03	0.9
Hyperactivity deficit symptoms	−0.18	0.35	−0.39	0.2	−0.51	0.06
Global symptoms composite score	−0.23	0.25	−0.35	0.27	−0.41	0.15
Behaviour regulation composite score	−0.22	0.29	−0.19	0.53	−0.02	0.95
General executive composite score	−0.28	0.16	−0.34	0.29	−0.13	0.66

* *P* < 0.05.

** *P* < 0.01.

## Discussion

The present study was motivated by the lack of controlled studies on the effect of cognitive stimulation treatments over both the clinical-cognitive symptoms and the neurophysiological correlates of the disorder. Based on the existent literature on electrophysiological changes associated with cognitive treatments across different pathologies, including ADHD, we decided to focus on modulations of alpha power, putatively induced by a digital cognitive intervention in which cognitive load was progressively increased under supervision of a CBR algorithm. These sorts of algorithms leverage information from the user experience to feed and improve the system.^53^ Additionally, they do not require manual configuration of the parameters that govern the intelligent system. Finally, while they allow a global learning of how to increase the level of difficulty of the users, it is possible to individually adjust that pattern to the particular features of a given user.

Many studies have demonstrated that cognitive interventions are useful to improve many of the cognitive and behavioural symptoms of ADHD. However, whether digital versions of those treatments were equally effective was still under controversy.^54,55^ Two recent studies have shown that empirically based digital training could indeed be effective in reducing cognitive symptomatology of ADHD.^22,23^ Our findings are in line with this most recent evidence and provide further support to the use of this sort of intervention in a clinical context. Specifically, and in line with our *a priori* hypothesis, participants in the experimental group experience significant enhancement of oscillatory power in the alpha band. Moreover, this effect was associated with post-treatment changes in measures of attentional deficits (i.e. EDAH), suggesting a functional relationship between this neurophysiological marker and the behavioural measures of the disorder. Alpha power has been related to states of hyperarousal,^56,57^ working memory maintenance^5^ and inhibitory control,^6^ all of the core processes affected in ADHD.^7^ On the other hand, experiments focused on other neuropsychiatric conditions have reported neurophysiological changes induced by similar non-pharmacological interventions, for instance in acquired brain injury,^58^ alcoholism^26^ or even cancer.^59^ Thus, it seems reasonable that a therapeutic intervention that is effective at the cognitive level is also able to induce changes in brain dynamics associated with the core symptomatology of the disorder^60–64^; Rodriguez-Jimenez *et al*.^65–67^ Consistent with other studies, this increase in alpha power could be related to a decrease in hyperarousal^25^ in ADHD patients with high alpha power, thus reducing some of the core symptoms of TD.

Although we did not formulate hypotheses on the topography of the effect, our results were very much in accordance with previous reports that found similar alpha power increases located over posterior regions, mainly the parietal cortex.^68,69^ In particular, the post-training alpha-band power increase in the precuneus region is of major interest. This parietal region, in functional connection with the prefrontal cortex, has been shown to play a critical role in ADHD.^70^ Reductions of the functional coupling between the precuneus and the anterior cingulate cortex have been previously reported in ADHD.^71^ Besides, lower short- and long-range connectivity in dorsal attention (superior parietal cortex) and default mode nodes (i.e. precuneus) has been reported,^72^ and proposed to reflect part of the underlying deficit in working memory performance that is a hallmark of this condition.^73^

Additionally, the cluster that concentrated the significant differences in alpha power also extended over central regions, including the precentral and postcentral gyri. Meta-analytic evidence has shown that motor excitability is a reliable feature of ADHD,^74^ as an important feature of a more general deficit of response control.^75^ Since oscillations within the alpha band are widely accepted to reflect cortical inhibition,^6^ the power enhancements in these central areas observed in the experimental group might well reflect an improvement in the level of cortical excitability associated with impulsive behaviours, putatively induced by our cognitive intervention. Unfortunately, our study did not include specific measures of motor impulsivity, so this relationship remains speculative until new studies including such measures are conducted.

Altogether, our results provide further evidence on the efficacy of digital cognitive interventions for ADHD, particularly when they are empirically based and they include increases in cognitive load individually adjusted. Also, the MEG findings suggest that this type of intervention can induce changes in some of the oscillatory dynamics of brain function, particularly the power density of the alpha band. More interestingly, the functional relationship between post-treatment alpha power enhancement and improvements in inattentive symptomatology suggest that the intervention here proposed is able to trigger changes at two interrelated levels.

These results should be considered carefully since the study was not exempt from limitations. The most important one is in regard to the size of the sample involved. Although small sample sizes can affect the statistical power of the analysis, groups between 10 and 15 participants are well-supported in similar literature.^58,76,77^ Also, our study did not include a sample of normotypic healthy participants that would have provided valuable information to understand the specific mechanisms by which the intervention seems to be effective. Therefore, at the light of the results and limitations derived from the study, we have decided to run a pivotal study with a randomized, double blinded, parallel clinical trial design that will account for the present shortcomings. First, the planned sample size will be increased to ensure statistical power enough to detect smaller treatment effects. Second, and based on the results here reported, the study will be confirmatory in regard with both the frequency band of interest, and the parietal topography of the effect as well as its relationship with other cortex areas such as prefrontal cortex.

In conclusion, the present work provides novel evidence on the effectiveness of digital treatments based on progressive increases in cognitive load, adjusted by AI algorithms, as well as their putative neurophysiological underpinnings.

## Supplementary Material

fcac038_Supplementary_Data

## Abbreviations

ADHD =: attention deficit hyperactivity disorder
AI =: artificial intelligence
CBPT =: cluster-based permutation test
EOG =: electrooculogram
HPI =: head position indicator
MEG =: magnetoencephalography
MNI =: Montreal Neurological Institute
