# Supplementary material for: Alpha-band power increases in posterior brain regions in attention deficit hyperactivity disorder after digital cognitive stimulation treatment: randomized controlled study
Source: Brain Commun. 2022 Feb 17;4(2):fcac038. doi: 10.1093/braincomms/fcac038 (PMC8984701; doi:10.1093/braincomms/fcac038)
Supplement: fcac038_Supplementary_Data [file fcac038_supplementary_data.zip › Supplementary Material 1.docx]

# Supplementary Material 1. Complementary information about methodology and materials

## Experimental design

This is a single-center, parallel, single-blind, randomized, controlled study aimed to paediatric population (8-11 years) diagnosed with ADHD of combined presentation.

Participants were randomly allocated in one of the two independent conditions: Experimental group (KAD_SCL_01® intervention) or Control group (Sham control intervention). Baseline assessment for each participant was performed before the at-home intervention period (pre-intervention assessment). After that, both conditions received an at-home intervention (Experimental group received KAD_SCL_01® and Control group received Sham control) for 12 weeks. Identical assessment was performed once at-home intervention period was over (post-intervention assessment). Because all participants were administered with the same neuropsychological assessment battery, potential practice effect remained constant to all of them.

Study procedure is shown as follows:


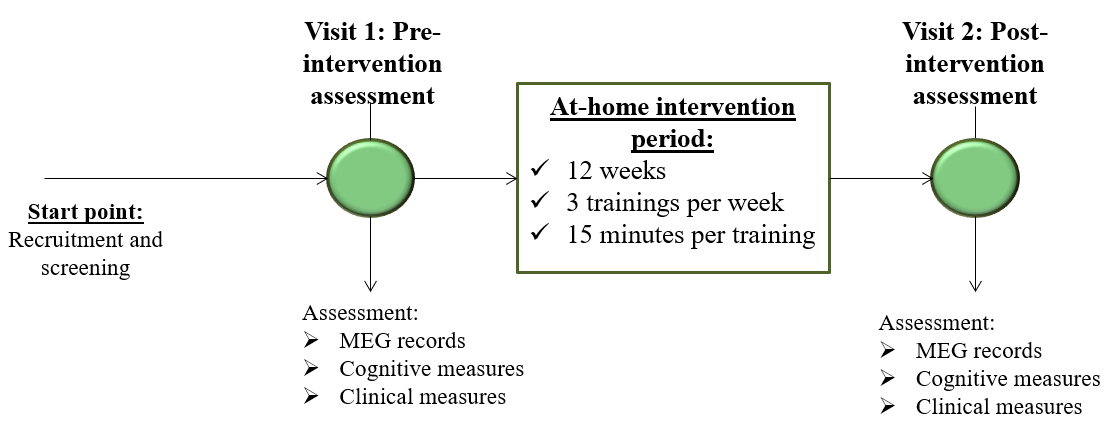


Supplementary Figure 1: Study Procedure

## Digital intervention

***Experimental condition. KAD_SCL_01®*** ***cognitive intervention*.**

Participants allocated in the Experimental condition carried out KAD_SCL_01® cognitive intervention. KAD_SCL_01® is a stand-alone software which generates a complete cognitive intervention based on fourteen neuropsychological treatment *videogames*.

Digital cognitive intervention with KAD_SCL_01® software consisted in 36 cognitive training sessions. Each cognitive training session is 15 minutes long and consisted in a pack of three KAD_SCL_01® games which changed daily. Training sessions were scheduled as follows: three 15-minutes intervention sessions per week for 12 weeks. One session per day was allowed only. Intervention sessions were performed at home, with no supervision or support from a therapist or family member, on a tablet with an Android (ver. 4.0.3 and higher) or iOS (iOS 8.0 or later) operating system. Intervention sessions were monitored telematically for frequency and duration daily by study technicians. Monitoring protocol was as follows: participants and legal guardians were instructed to perform three sessions per week. If at the 4th day of each week of the intervention they did not comply with at least one scheduled session, the study technician made a phone call to participant’s legal guardian/s.

Each KAD_SCL_01® game is designed to work on different cognitive processes with an increase of the cognitive load following evidence that the brain’s reconfiguration networks seem to be fixed by this type of training routine. The fourteen games which compose KAD_SCL_01® cognitive intervention are described in the Annex S2 from the current Supplementary Material in terms of game dynamics, integrated cognitive processes and hierarchical or multi-level structure (game modes).

Game level is adapted based on case-based reasoning algorithm. This algorithm compared the performance and clinical features of the patient with a huge data base of patients in each cognitive training season in order to fit the best game level. The aim of this game level calculation is that patients always performance in a range of 40% - 70% of success in the game. So, in a simplified way, it can be said that when patient’s performance above these range the game level will increase, and when the patient’s performance below these range the game level will decrease. The algorithm and all the specifications, as well as the human-AI interaction regarding with the algorithm, are described in the Annex S3 from the current Supplementary Material.

***Control condition. Sham intervention.***

Participants in the Control arm received a sham intervention. This sham intervention was composed by three videogames which are not specifically designed in order to improve cognitive performance, following Mishra, Anguera and Gazzaley’s (2016) criteria.

These videogames were Knightmare Tower, Bloons Super Monkey and Super Staker 2. Knightmare Tower is a runner-like videogame in which the player has to ascend to the top of a tower while avoiding enemies and traps. Bloons Super Monkey is a videogame, similar to the classic Space Invaders, in which the player has to defeat enemies and obstacles by moving left or right. Last, Super Stacker 2 is a puzzle-like videogame in which the player has to locate a certain number of geometrical pieces in order to keep them balanced. Sham intervention tasks are accessible through Kongregate open-access platform (https://www.kongregate.com/).

In order to ensure sham intervention appeared as a legitimate intervention, it was prescribed under the same conditions as KAD_SCL_01® intervention. Participants had to perform 36 15-minutes sham intervention sessions for 12 weeks (3 sessions per week). Legal guardians were also asked for report any adverse event and compliance was checked daily (sham platform allows to check if participant played the day before). Kongregate platform also works with access credentials, like KAD_SCL_01®.

## Complete neuropsychological assessment

Participants received a complete neuropsychological assessment composed by the following standardized cognitive tests:

Conners’ Continuous Performance Test-III (CPT-III): CPT-III is a computerized continuous performance task which measures several aspects of attention and executive functioning. In this version, stimuli (14 letters; letter X is the target and the others are the distractors) are presented individually in the laptop screen. Participants have to press the space bar each time a distractor appears in the screen and inhibit this response when a target is shown. The task is composed by 360 trials, grouped by 8 blocks with variable inter-stimuli interval (ms) for each block.

Developmental Neuropsychological Assessment-II (NEPSY-II): NEPSY-II is a standardized neuropsychological battery, widely used in order to assess a vast range of cognitive processes, along the neurodevelopment. Psychometric properties have been published within the Spanish adaptation, including reliability, internal consistency, test-retest stability and internal and external validity. The following subtests were administered.

- Auditory Attention and Cognitive Flexibility Test. This test is based on an auditory detection task in which participants listen to a sequence of words from a recorded audio tape. Participants have to point the target in a piece of paper every time they listen to it. Targets are colors, and they are presented in a piece of paper with four colored circles (red, green, blue and black). In Auditory Attention, target word is “RED” (“ROJO” in Spanish). Every time the participants listen to the word “ROJO” from the audio tape, they have to point the red circle in the piece of paper. In Cognitive Flexibility condition, there are three target: “RED”, “GREEN” and “BLUE” (“ROJO”, “VERDE” and “AZUL” in Spanish). When they listen to the word “RED”, participants have to point the green circle, and, on the contrary, when they listen to the word “GREEN”, they have to point the red circle. In addition, when participants listen to the word “BLUE”, they have to point the blue circle.
- Verbal Fluency Test. This is a classical verbal fluency task in which participants have to verbally declare as many words as they are able to, following a certain condition, in 1 minute per condition. There are four conditions in total. These conditions are classified in Semantic and Phonological. In Semantic section, conditions are words which belong to the semantic categories of Animals and Food. In Phonological section, conditions are words which start with the letter P and with the letter M.
- Card Classification Test. In this test, a pack of eight cards with pictographic representations of animals in their natural habitats are given to the participant. The objective is to perform sequential classifications for these cards in two groups (4 cards per group) according with a classification rule which must be inferred by participant. There are 10 possible classification rules (e.g the size of the animals, the weather, the color of the card).
- Inhibition Test. Inhibition Test from NEPSY-II is a Stroop-based task in which a sequence of 40 black and white geometrical shapes (circles and squares) is presented in paper. Participants have to identify each item from the sequence by saying in out loud “CIRCLE” or “SQUARE” (“CÍRCULO” or “CUADRADO” in Spanish) as faster as they are able to, under three different conditions. First, as the items appear in the sequence; second, exchanging the shapes (circles as squares and squares as circles); and third, naming the black figures as they are shown and the white figures exchanging its identification (white circles as squares and white squares as circles).

Wechsler Intelligence Scales for Children-IV (WISC-IV): WISC-IV is a cognitive standardized battery which aims to measure different aspects of cognitive processing. Psychometric properties have been published within the Spanish adaptation, including reliability, internal consistency, test-retest stability and internal and external validity. The following subtests were administered.

- Digit Span Test. In this test, participants are asked to verbally repeat a sequence of digits, whose length goes increasing by one integer after each pair of trials, starting from a sequence of two digits. Two conditions are administered: forward (forward repetition of the sequence of digits) and backward (backward repetition of the sequence of digits). The administration of each condition ends after three consecutive wrong answers. This test is designed in order to measure verbal short-term and working memory.
- Symbol Search Test. Symbol Search is a test in which participants have to determine if one of the two target symbols is hold in a sequence of symbols. If there is at least one target between the sequence, they have to mark “YES” in the item’s answer box; else, they have to mark “NO”. Participants have to solve as many items as they are able to in two minutes. This test intends to measure rapid processing and visuospatial attention.
- Digit Symbol Substitution Test. In this test, the nine integers from 1 to 9 are paired to different symbols. These matches compound the substitution guide. The test consists of a sequence of 126 integer numbers (from 1 to 9) in which participants have to write below each integer its paired symbol following the substitution guide. They have two minutes to fulfill as many integers as they are able to.

Corsi Block Tapping Test from Wechsler Non-Verbal Scales (WNV): This test is a compound of the WNV battery and intends to measure spatial working memory. Stimuli are 9 equal blue cubes (or blocks) on a wood table. As in the Digit Span Test, evaluator performs a sequence by pressing the blocks in an order determined by test instructions and participants have to repeat this sequence (each sequence composes one trial). The length of the sequence increases by one block after each pair of trials. Two conditions are administered: forward (forward repetition of the block sequence) and backward (backward repetition of the block sequence). The administration of each condition ends after three consecutive wrong answers. From this test, number of correct answers in each condition (Total Forward and Total Backward) were computed. Span scores (length of the last sequence successfully repeated) for each condition (Forward Digit Span and Backward Digit Span) were also taken.

In addition, Clinical questionnaires about ADHD behavioral symptoms and executive functioning in daily activities compose the clinical secondary outcome measures section.

Behaviour Rating Inventory of Executive Function, Parent Version (BRIEF): BRIEF is an 86-item questionnaire which assess executive functioning in daily-life. It rates different behaviors which are related with executive function disorders from 1 to 3 according with its frequency and it is widely administered in ADHD assessment. BRIEF has been adapted to Spanish infant population with reliable psychometric properties and criterion validity with EDAH questionnaire.

Evaluation of Attention Deficit and Hyperactivity Disorder (EDAH): This questionnaire is a Spanish adaptation of the Conners’ Rating Scales with reliable psychometric properties. It is aimed to measure pediatric ADHD symptomatology (inattention, hyperactivity, behavioral disorders).
