# Supplementary material for: Alpha-band power increases in posterior brain regions in attention deficit hyperactivity disorder after digital cognitive stimulation treatment: randomized controlled study
Source: Brain Commun. 2022 Feb 17;4(2):fcac038. doi: 10.1093/braincomms/fcac038 (PMC8984701; doi:10.1093/braincomms/fcac038)
Supplement: fcac038_Supplementary_Data [file fcac038_supplementary_data.zip › Supplementary Table 1.docx]

| Supplementary Table 1. Details about MEG analyses' dropouts | | | |
| --- | --- | --- | --- |
| Number | | Reason | Relation with intervention |
| Experimental | Control |  |  |
| 0 | 3 | Abandonment of the study before intervention started | No |
| 1 | 1 | Technical issues (tablet malfunctioning and/or lack of Internet conection for an extended period of time) | No |
| 0 | 3 | Uncompliance with intervention protocol and lack of engagement | Yes |
| 2 | 1 | Low quality of MEG recordings | No |
| 1 | 1 | Other inconveniences unrelated with the study | No |
