# Supplementary material for: Alpha-band power increases in posterior brain regions in attention deficit hyperactivity disorder after digital cognitive stimulation treatment: randomized controlled study
Source: Brain Commun. 2022 Feb 17;4(2):fcac038. doi: 10.1093/braincomms/fcac038 (PMC8984701; doi:10.1093/braincomms/fcac038)
Supplement: fcac038_Supplementary_Data [file fcac038_supplementary_data.zip › Supplementary_Material_2.docx]

# Supplementary Material 2. Dynamics, cognitive processes integrated and hierarchical or multi-level structure of Sincrolab’ games

**Jupertino**

It is one-game mode game: A series of moving stimuli ('fireballs') will appear and the objective of the game is to click a button ('catch') at the moment when the stimuli pass through the circle ('portal') of the same colour of the stimulus.

This game integrates visual tracking, visuomotor coordination and inhibitory control. As cognitive load increases, the speed of the trajectories is faster, so speed processing is progressively more relevant in cognitive performance.

Supplementary Figure 2: Game mode 1


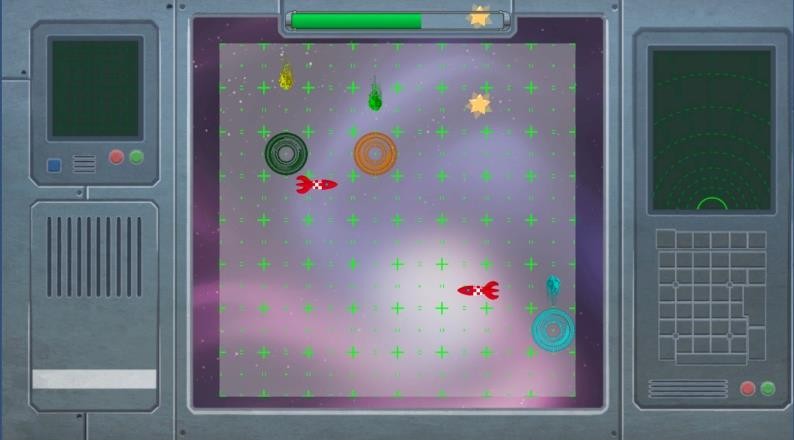
*
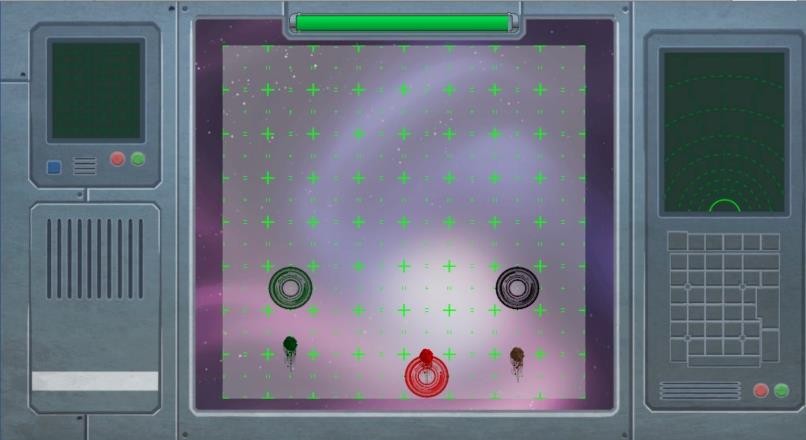
*

**Albeldum**

It is two-game modes game: labyrinths are presented on screen and the objective Sis to find the right way among multiple paths, according with determined conditions, changing throughout sessions. The second and more complex game mode includes numerical weights for each path in multitasking condition.

This game integrates planification, alternative response generation and inhibitory control. In addition, each labyrinth has a response period, so speed processing is also integrated. Therefore, the multitasking game mode recruits numerical working memory and calculus processes.

*Game mode 2*

*Game mode 1*


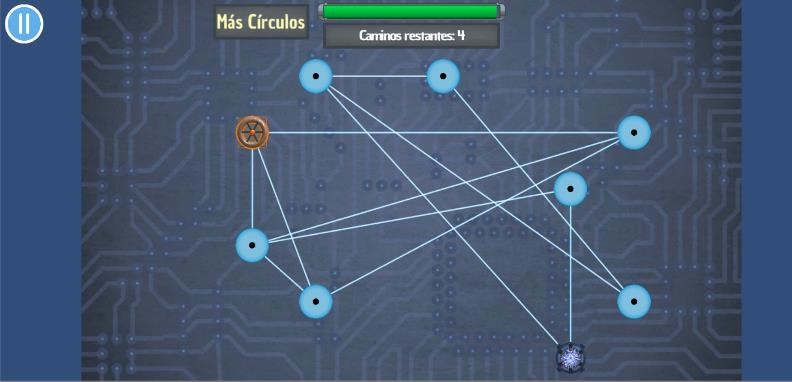

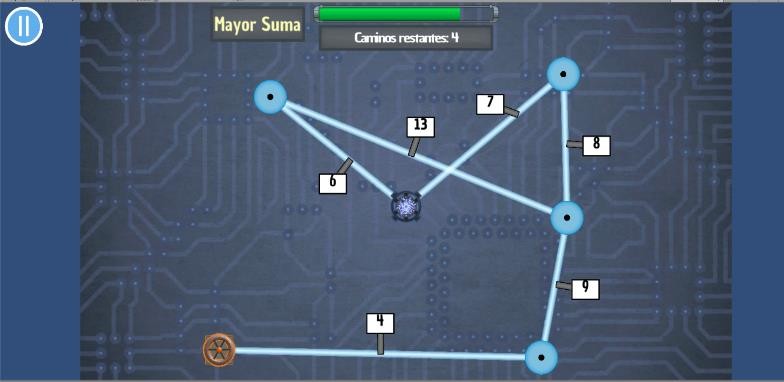


Supplementary Figure 3: Game mode 1 and 3

**Atomux**

It is seven-game mode game: according to an established classification rule, subjects should order the stimuli ('extraterrestrials') that appear. In the consecutive game modes different features are incorporated in the models, changing of rules between sessions or in the session, one of the models could disappear and/or a task of deduction could be necessary to resolve it.

This game is a categorization task that integrates the training of selective attention and executive functions, specifically cognitive flexibility, problem solving, inhibitory control and decision making.

*Game mode 4*

*Game mode 1*


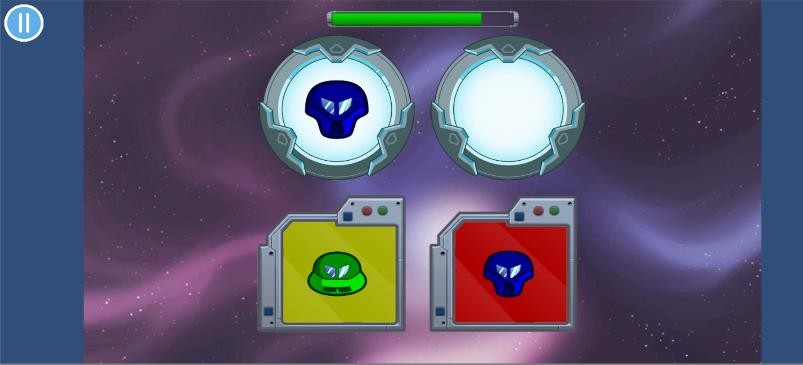

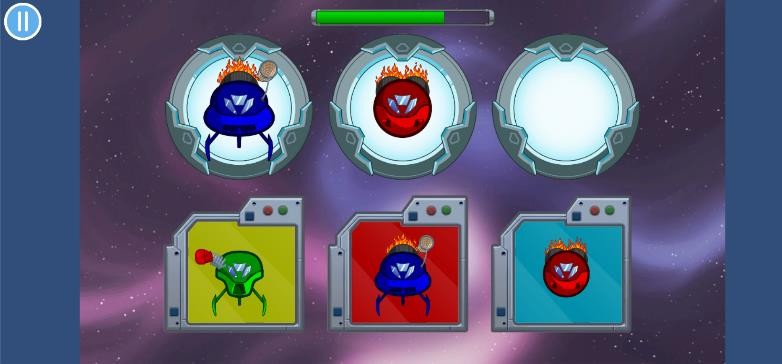


Supplementary Figure 4: Game mode 1 and Game mode 4

**Storms**

It is a thirteen-mode game. They include exercises of adding, counting, repeating, ordering, and sequencing stimulus. In consecutive game modes the different exercises can be combined to create a task switching exercise.

This task is intended to train alternate attention, visuospatial operating memory, visuospatial short loop memory, calculation and mathematical logical reasoning.

Supplementary Figure 5: Game mode 3 and Game mode 11

*Game mode 11*

*Game mode 3*


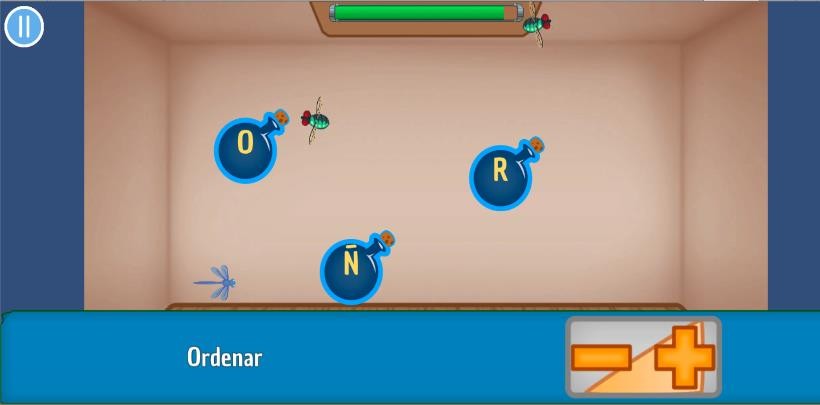

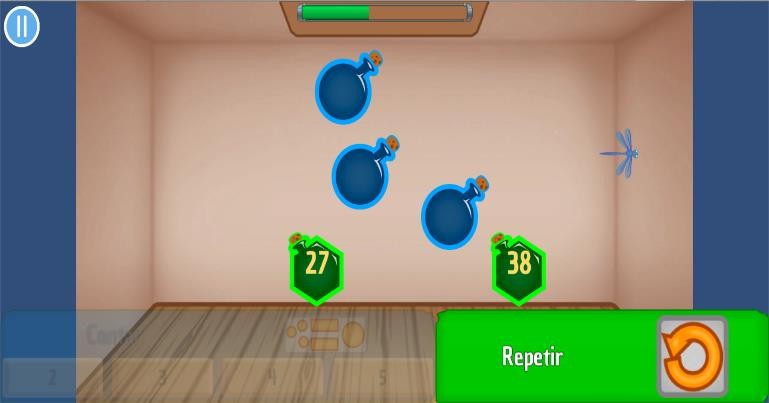


**Isia**

It is seven-game mode game: The objective is to click when a target stimulus appears ('samurais', 'evil doctor', 'bears', etc...) in different conditions. Sometimes distracting stimuli appear which must be inhibited and/or there are signs to warn participants to suppress initiated responses.

This game is an inhibitory control, sustained attention, processing speed, and decision-making task. Task switching using different rules inside the session allows the stimulation of cognitive flexibility. In this game different changes among the visual and auditory path occur too.

*Game mode 1*


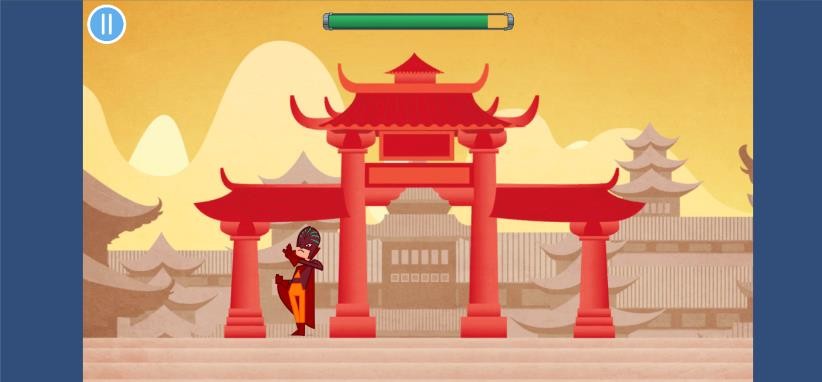

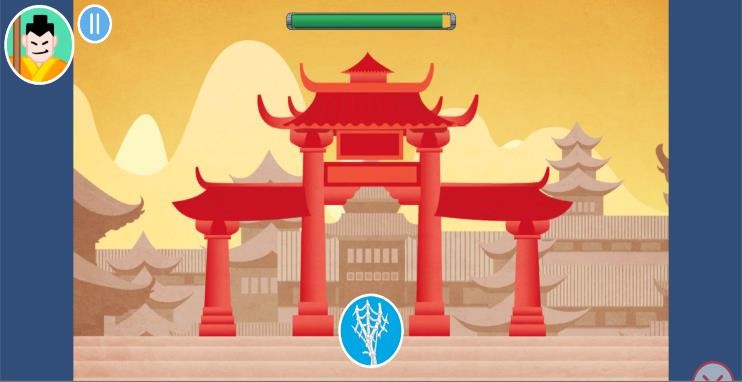


*Game mode 4*

Supplementary Figure 6: Game mode 1 and Game mode 4

**Cratero**

It is a five-game mode game: A series of guides or models ('skewers' or 'totems') appear and the subject must manage to copy them in other empty guides. The goal is to fill as many skewers or totems as possible according to the different instructions and activities offered. In the consecutive game modes rules about the order of copy are included and/or the models could disappear and / or other stimuli may appear which, when pressed, give them more points or others that can remove it.

This game integrates different cognitive processes such as planning, generation of alternative responses, logical- mathematical reasoning and processing speed. The multitasking game modes also integrate inhibitory control and/or working memory.

*Game mode 4*

*Game mode 2*


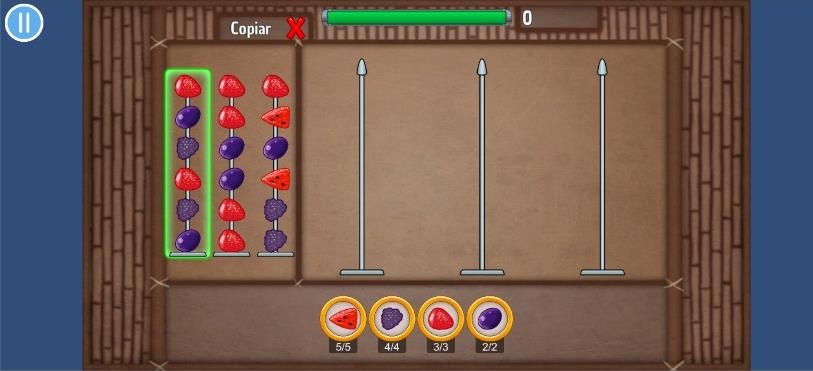

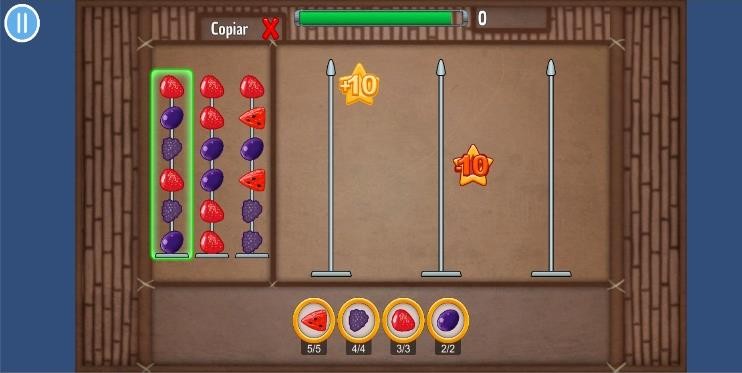


Supplementary Figure 7: Game mode 2 and Game mode 4

**Bublos**

It is four-mode game: the participants have to solve a puzzle fitting different geometric shapes

This game integrates visuospatial functions, mainly perception, orientation, and visuospatial localization.

*Game mode 3*

*Game mode 1*


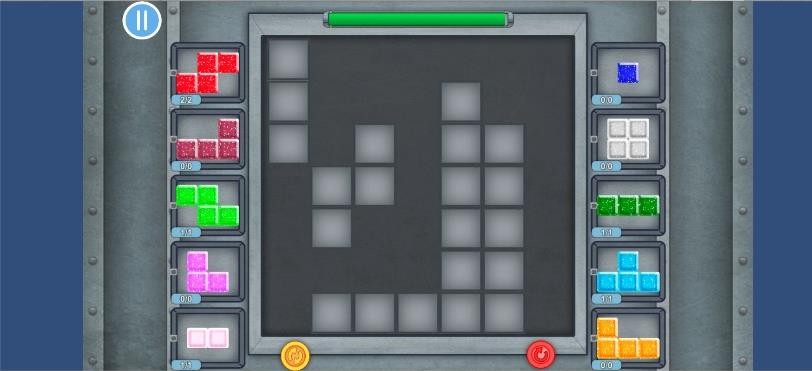

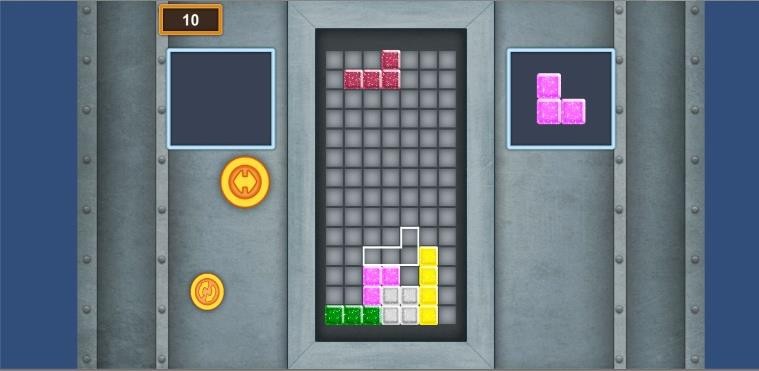


Supplementary Figure 8: Game mode 1 and Game mode 3

**Redos**

It is a one-mode game which consists of locating the opponents' cards that are equal to the player's according to a set category. At the top level of the game a task of deduction could be necessary to solve it.

This game integrates selective attention, sustained attention, processing speed, decision making, inhibitory control, alternating attention and working memory.

*Game mode 1*


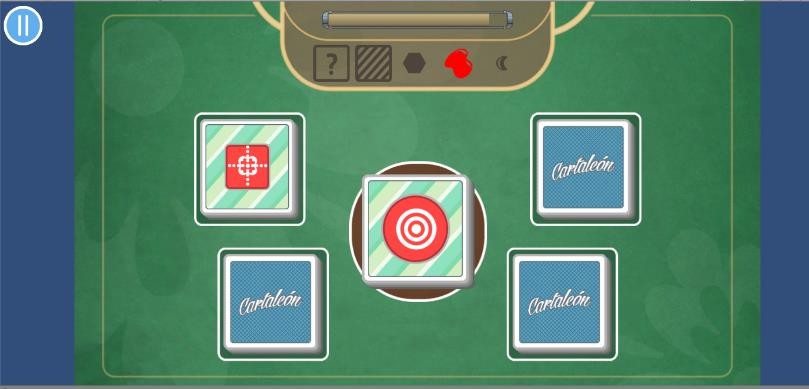

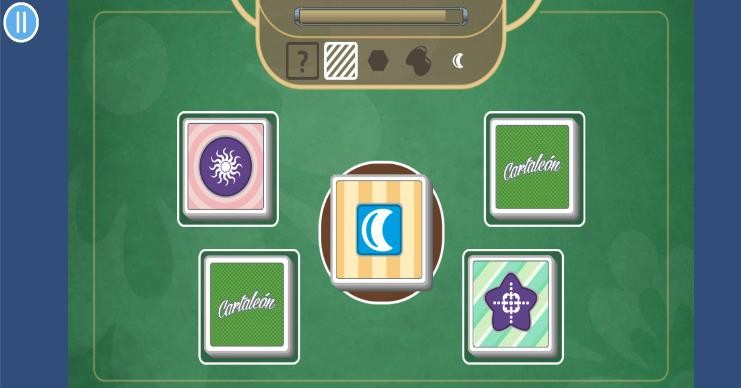


Supplementary Figure 9: Game mode 1

**Nublox**

It is a three-mode game consisting in memorize pairs or trios of cards which could be based on images, numbers, colours or words. The relation among the cards sometimes is congruent and others incongruent. Throughout the game modes multiple options allow this game to encourage calculation, phonological and lexical path stimulation.

This game integrates processing speed, short-term memory and working memory.

*Game mode 2*

*Game mode 1*


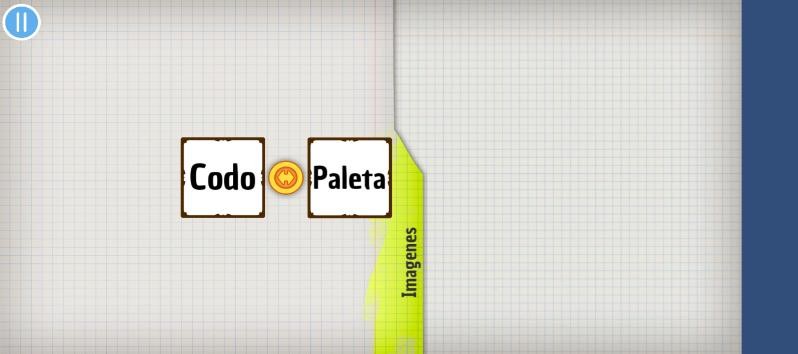

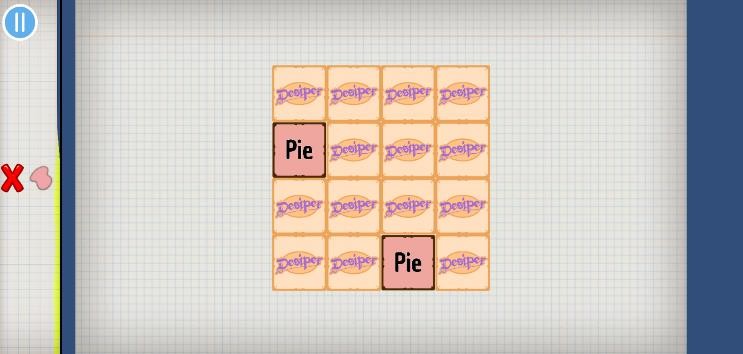


Supplementary Figure 10: Game mode 1 and Game mode 2

**Lunar**

It is a seven-mode game: different type of stimulus in visual or auditory paths appear and the participant must respond when the stimulus is repeated immediately after or when another different stimulus appears between the first and second or first and third appearance of the target stimulus. In the consecutive game modes changing of rules between sessions or in the session are included and multitasking in same or different sensory path could incorporated.

This game integrates working memory, sustained, alternate, divided attention, processing speed and inhibitory control across task switching and multitasking.

*Game mode 5*

*Game mode 3*


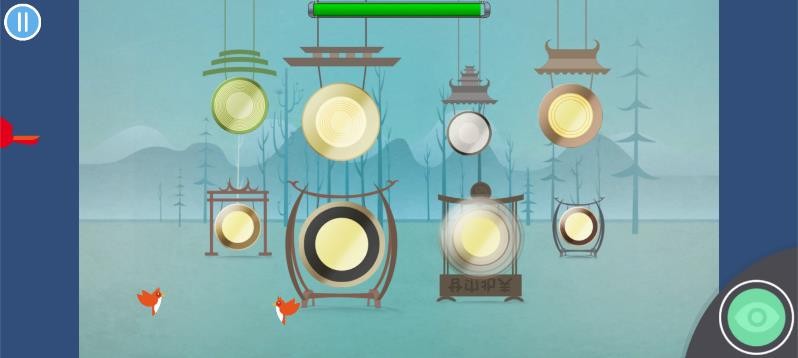

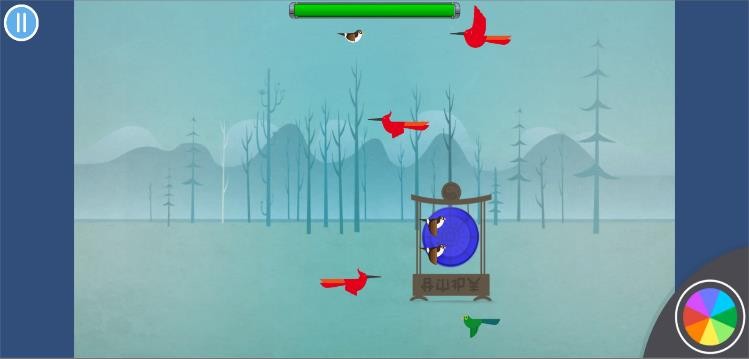


Supplementary Figure 11: Game mode 3 and Game mode 5

**Akua**

It is a five-mode game: in this game the participants drive a car in a zenith view and they should avoid colliding with other cars. In consecutive game modes this task is performing in multitasking with calculation, logic, language, memory and inhibition tasks.

This game integrates sustained, divided attention, processing speed, inhibitory control and reasoning.

*Game mode 5*

*Game mode 2*


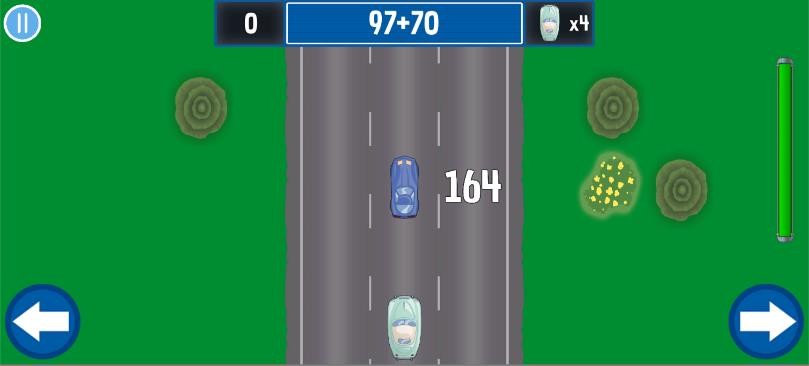

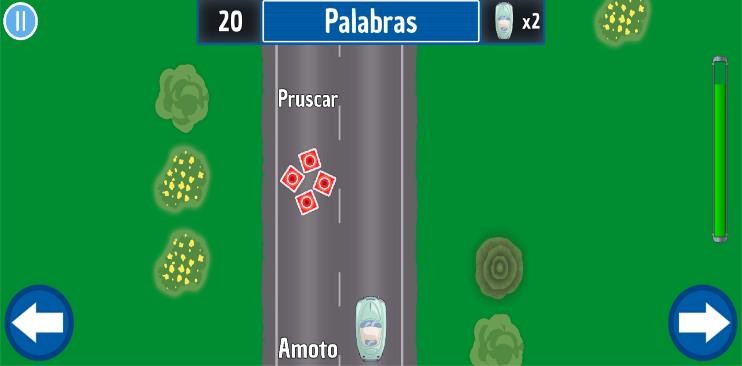


Supplementary Figure 12: Game mode 2 and Game mode 5

**Gaia**

It is a two-mode game: participants must maintenance and update visual material. Different stimuli ('planes' or 'dragons') will appear, assigned to different containers ('islands'). The task is to keep track of all the stimuli that are hidden behind each container. In the second game mode different stimuli enter, exit or are exchanged between the containers forcing a continuous update of the material stored.

This game integrates working memory, update processes and selective attention.

*Game mode 2*

*Game mode 1*


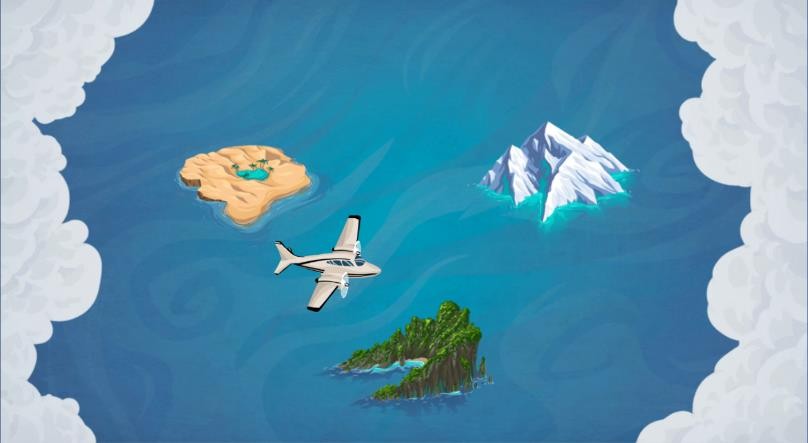

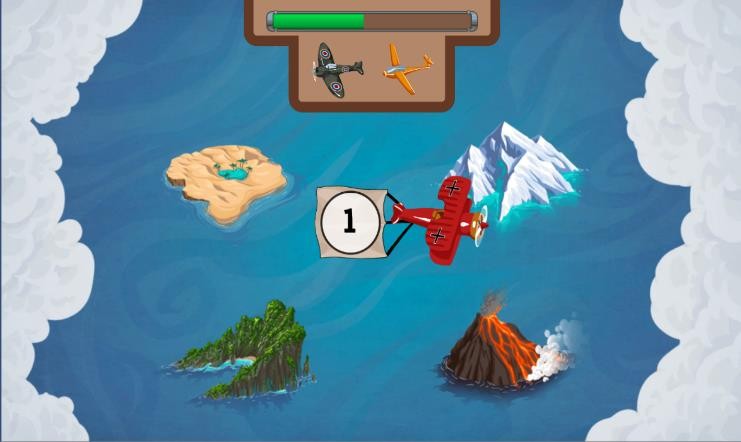


Supplementary Figure 13: Game mode 1 and Game mode 2

**Tronic**

It is five-mode game: Tronic is a problem-solving and planning task in which the objective is to find out a secret code through multiple attempts. In the different game modes, it can be needed to solve the code from the beginning, with or without repeated elements or answered questions about it about updates of the code.

This game integrates working memory, planning, problem solving, and decision making.

*Game mode 4*

*Game mode 1*


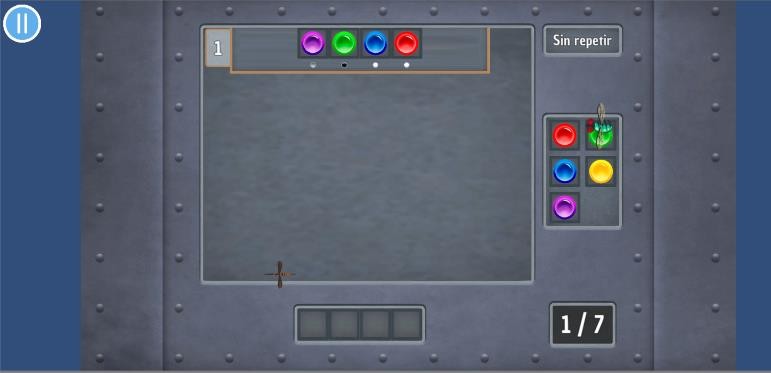

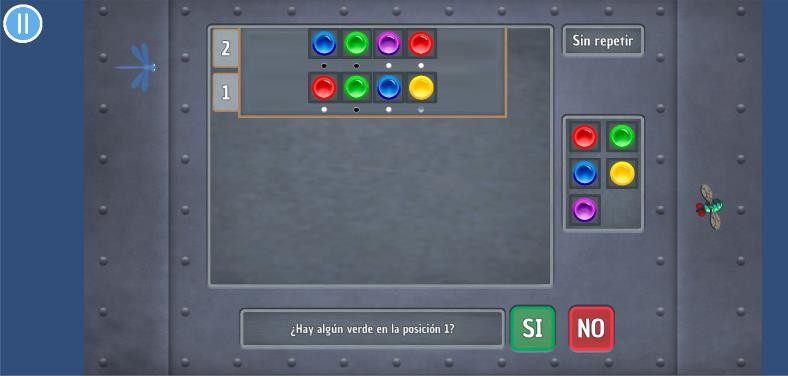


Supplementary Figure 14: Game mode 1 and Game mode 4

**Anium**

It is a six-mode game which a series of stimuli will appear that will have to be sought within a stimulating field. In some game modes the participants need to find the similarities or differences between two complex stimulating fields, these can be organized or disorganized, a fixed pattern of searching will be required and/or the targets could vanish.

This game integrates selective and sustained attention, as well as visual tracking among, working memory and planification.

*Game mode 5*

*Game mode 2*


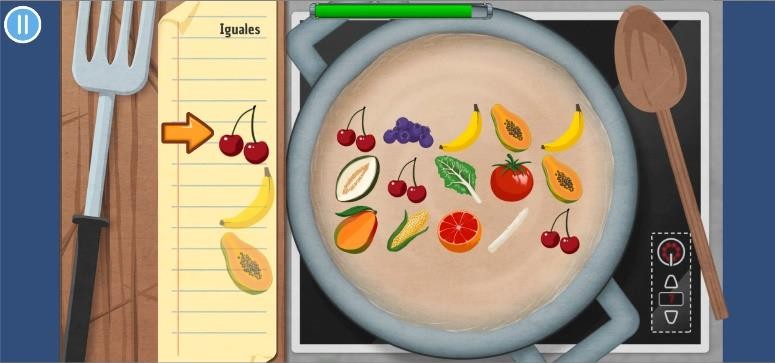

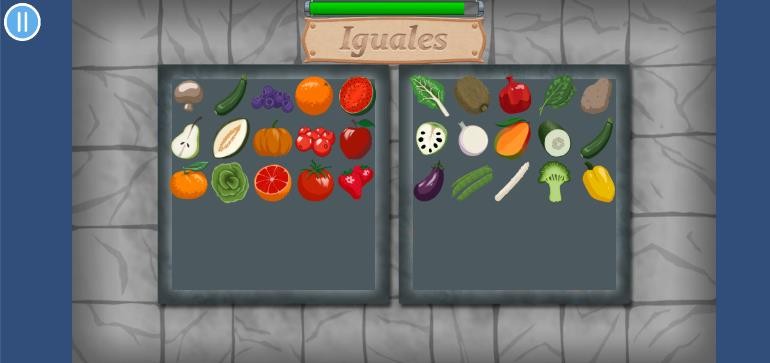


Supplementary Figure 15: Game mode 2 and Game mode 5
